# Editorial: Reviews in the neuroscience of orofacial functions

**DOI:** 10.3389/froh.2023.1304573

**Published:** 2023-10-30

**Authors:** Nikolaos Christidis, Nikolaos Nikitas Giannakopoulos

**Affiliations:** ^1^ Division of Oral Diagnostics and Rehabilitation, Department of Dental Medicine, Karolinska Institute, and Scandinavian Center for Orofacial Neurosciences (SCON), Huddinge, Sweden; ^2^ Department of Prosthodontics, University of Würzburg, Würzburg, Germany; ^3^ Department of Prosthodontics, Dental Faculty, National and Kapodistrian University of Athens, Athens, Greece

**Keywords:** systematic review, temporomandibular disorders (TMD), orofacial pain, endodontics, bruxism, treatment

**Editorial on the Research Topic** Reviews in the Neuroscience of Orofacial Functions

With the ever-increasing number of publications in the field of modern medicine and the fact that evidence-based medicine is becoming one of the most important components in health education as well as in the daily clinical practice of health professionals (1–4), it has begun to be challenging, almost impossible, for educators, researchers, and busy health professionals to stay up to date with the existing primary research. With the help of reviews, existing information can be summarized and evaluated regarding quality and bias, and narrative, qualitative, or meta-analytic data can be provided to help students, health professionals, and researchers improve reliability and accuracy, i.e., what works and what does not work (5, 6).

Neuroscience is a multidisciplinary scientific discipline combining physiology (including neurophysiology and electrophysiology), anatomy, molecular biology, developmental biology, cytology, psychology, odontology, and medicine, as well as physics, chemistry, and computer science (7, 8). Research in the field of neuroscience aims to provide knowledge to understand the role and development of the nervous system as well as the etiology, pathology, diagnostics, treatment, and prognostics of orofacial neural and musculoskeletal disorders (7, 9). In the orofacial field, neuroscience investigates oral functions such as mastication and swallowing, as well as conditions or disorders that can occur during sleep or when a person is awake, e.g., apnea and bruxism. Another major research branch within the field of orofacial neuroscience handles all aspects of orofacial pain from epidemiology to basic mechanisms, diagnostics, treatment, etc.

Based on this, the present Research Topic aimed to highlight recent advances in the field, while emphasizing important directions and new possibilities for future inquiries. The anticipation was and is that the research presented will promote discussions among students, health professionals, and researchers in the field of neuroscience that in the long run will translate to best practice applications in clinical, public health, and policy settings.

This Research Topic consists of four published manuscripts, one bibliometric analysis, and three systematic reviews, involving 17 different authors spread around the globe. Al-Sharee and co-workers performed a bibliometric analysis to identify and analyze the top 100 cited publications in the field of temporomandibular disorders (TMD) in order to guide any professional level with an interest in this topic by mapping the current trends in the field of TMD. Among 8,927 publications from the year 2000 onward, the top cited paper was a study on the diagnostic criteria for TMD, with 1,287 citations published in 2014 in the Journal of Oral and Facial Pain and Headache. This journal also had most of the top 100 cited publications. All publications among the top 100 cited were considered classic, i.e., having more than 100 citations each. Eighty-one percent of the most cited studies were from the USA and Europe. Since 2000, researchers in the field of TMD have mainly shown interest in and published papers on (a) diagnostic criteria; (b) TMD symptoms and mainly pain-related symptoms; (c) etiology and risk factors of TMD and mainly bruxism; and (d) the treatment of TMD. On the other hand, research topics such as imaging, occlusion, tissue engineering, and disk displacements are presently not as prominent. Finally, 33% of the included studies were review articles.

Restrepo-Serna and Winocur aimed to systematically review the literature on bruxism in children with the aim of compiling the best available evidence. Literature searches in the databases PubMed, Medline, EBSCO, SCOPUS, and Google Scholar were performed to identify all studies on humans published from 2015 to 2023, assessing the genetic, biopsychosocial, and sleep factors associated with any different approaches for sleep bruxism (SB) in children and their interventions. Out of 171 identified studies, 16 were selected and included in this Systematic Review. Based on the included studies (including self-report, clinical, and instrumental bruxism assessments), there were positive associations between (a) alterations in sleep behaviors and architecture and (b) sleep breathing disorders with (1) genetics, (2) quality of life aspects (school and emotional functions and overuse of screen time), (3) mother anxiety and family conformation, and (4) diet. Furthermore, the included literature indicates that there seems to be possibilities to increase airway patency and through this reduce the occurrence of SB. Tooth wear, on the other hand, was not found to be a major sign of SB in children. However, methods of SB assessment are heterogeneous and hamper a reliable comparison of the results.

Shimada and co-workers aimed to systemically review the literature on the effectiveness of exercise therapy on pain relief and jaw mobility in patients with pain-related TMDs. Literature searches in the databases PubMed, Web of Science, Cochrane Library, Ovid, EBM reviews, and Academic Search Complete were performed to identify studies published from the beginning of each database until March 2022 on the following two focus questions: (a) is exercise therapy effective at reducing clinical pain intensity in patients with painful TMD compared with the control group investigated by randomized controlled trials (RCTs); and (b) is exercise therapy effective at improving jaw movements in patients with painful TMD compared with the control group investigated by RCTs? Out of 3,388 identified studies, only five (with a total of 145 participants) were eligible for inclusion. Owing to the heterogeneity and small number of participants in the included studies, a meta-analysis could not be performed. Therefore, data were synthesized according to synthesis without meta-analysis (SwiM). This synthesis suggests that exercise therapy, especially coordinate exercise, can be effective in managing painful TMD conditions, as among the included exercise modalities, only the coordination exercise showed a significant effect on pain relief and the improvement of joint mobility. However, further research is needed to establish optimal parameters for this patient population, as well as standardization and consistency in terminology and treatment structure.

Finally, Alhilou and co-workers aimed to investigate the present knowledge on postoperative pain related to the two types of emergency treatments available for treating symptomatic irreversible pulpitis (SIP) and symptomatic apical periodontitis (SAP). Literature searches in the databases Medline (Ovid), Embase, and Web of Science were performed to identify clinical controlled trials investigating the use of emergency pulpotomy and/or pulpectomy to alleviate pain (toothache) in SIP and SAP, published from the 1st of January 1978 until the 31st of December 2022. Out of 4,578 identified studies, only five studies were eligible for inclusion in this systematic review. The outcome of this narrative Systematic Review indicates that both pulpotomy and pulpectomy are suitable treatment options for SAP and SIP, as they provide sufficient pain reduction in the permanent dentition. However, this review cannot provide any conclusion whether pulpotomy or pulpectomy is to prefer since the results of the included studies were inconsistent. Furthermore, even though the included studies had a low risk of bias, none of the included studies accounted for essential and potential confounding variables, such as other factors affecting the patient perception of pain (including the psychological aspects). Additionally, possible non-odontogenic pain was not accounted for and could consequently affect the outcome of the included studies. Taken together, there are controversies within the available randomized control trials about which treatment is the most effective in reducing emergency pain in SIP and SAP. Thus, further well-designed studies are warranted.

Overall, this Research Topic highlights that a major part of the research in the field of orofacial neuroscience handles factors associated with intra- and extra-oral pain, mostly in the field of TMD. These aspects concern the diagnostics, pain-related symptoms, etiology, and risk factors of TMD with an emphasis on bruxism, and the treatment of painful conditions with an emphasis on TMD. It also highlights that even though there are thousands of studies performed, only a very few are eligible for inclusion in systematic reviews. However, the systematic reviews indicate that alterations in sleep behaviors and architecture as well as sleep breathing disorders are associated with biopsychosocial and dietary factors, while, interestingly, tooth wear does not seem to be a major sign of sleep bruxism in children. Furthermore, when it comes to treating the pain associated with orofacial conditions, this Research Topic indicates that exercise therapy, especially coordinate exercise, seems to be effective in managing painful TMD conditions, while both pulpotomy and pulpectomy seem to be equally effective in alleviating odontogenic pain, such as symptomatic irreversible pulpitis and symptomatic apical periodontitis.
